# Do national human resources for health policy interventions impact successfully on local human resources for health systems: a case study of Epworth, Zimbabwe

**DOI:** 10.1080/16549716.2019.1646037

**Published:** 2019-08-01

**Authors:** Bernard Hope Taderera

**Affiliations:** Department of Environmental Health, University of Johannesburg, Johannesburg, South Africa

**Keywords:** Decision space, healthcare worker reform, policy interventions, impact, Epworth

## Abstract

**Background**: The global health workforce crisis remains a challenge undermining health system strengthening in low-income peri-urban areas. Whilst the 2018 Astana Declaration and the 2030 Global Health Workforce Strategy are helping guide effort to address this challenge, the Decision Space Approach presents an opportunity through which to further understand decision space and its impact on innovation and performance, and what it can contribute towards the goal of health-care worker reform.

**Objective**: To use the Decision Space Approach to understand how national policy interventions on health workers impact local health-care worker systems in Epworth, Zimbabwe.

**Methods**: A case study design, within which cross-sectional studies were carried out at the principal and agent level, was used. At the principal level, data were collected through a documentary search and key informant interviews and generated a Human Resource for Health Policy Decision Space Mapping Analysis Conceptual Tool. The Conceptual Tool guided data collection at the agent level, where a documentary search, in-depth interviews and focus group discussions were carried out. The Tool facilitated discussion of findings and was complemented by interpretive thematic analysis and descriptive statistics.

**Results**: Intervention by the health ministry resulted in moderate decision space within which functional innovation, in partnership with the local board and church mission, revived financial budgeting, human resources planning, deployment, and retention. However, low capacity of the principal undermined the implementation of choices generated from narrow decision space in training, performance management, labor relations, safety, and information and research.

**Conclusions**: Whilst collaborative intervention by the principal may help revive health-care worker systems in low-income peri-urban areas, financial and technical incapacity of the principal and agent may undermine performance. Narrow decision space brings health-care worker reform policy direction but incapacity undermines progression towards universal health coverage and the Sustainable Development Goals in low-income peri-urban areas.

## Background

The global health workforce crisis characterized by an estimated shortage of almost 4, 3 million health-care workers particularly in Sub-Saharan Africa as reported by the World Health Organization (WHO) in 2006 remains a challenge undermining the pursuit of universal health coverage in low and middle-income communities [1]. To mitigate this challenge, the Global Health Workforce Alliance formulated the 2030 Global Strategy on Human Resources for Health, which has also been reinforced by resolution WHA67.24 on Follow-up of the Recife Political Declaration on Human Resources for Health of May 2014, and the 2018 Astana Declaration [2–5]. However, there has been inadequate research attention on the use the Decision Space Approach to understand how national health-care worker policy interventions impact local health personnel systems, and what it can contribute towards health-care worker reform in resource-constrained peri-urban areas [1,4].

**Figure 1.**
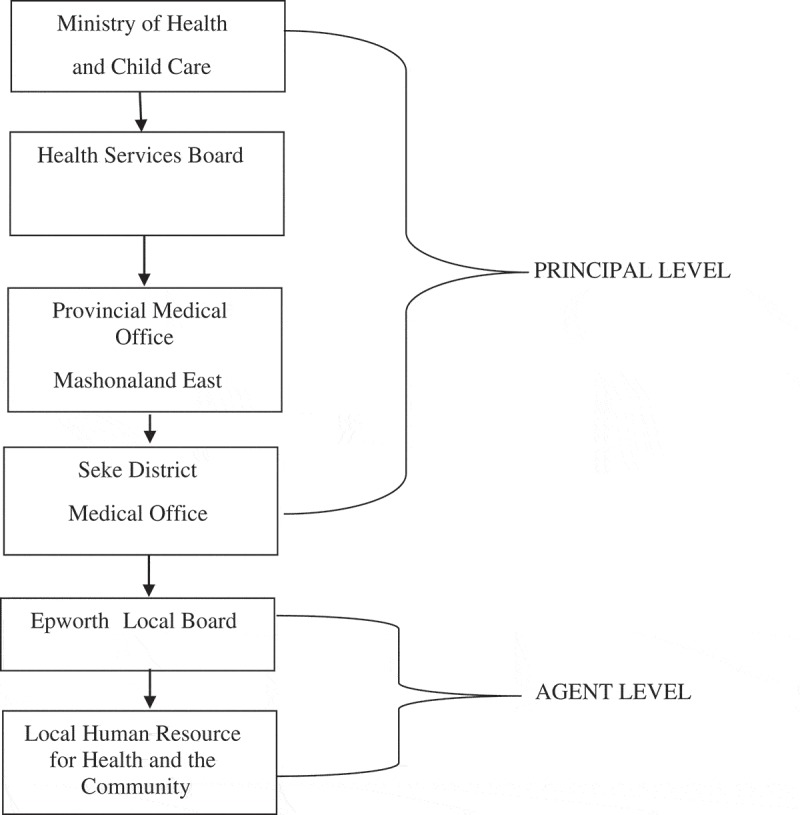
Levels of HRH decision-making. Reproduced with permission from BH Taderera et al. [1,4].

Human Resources for Health (HRH) reform can be traced back to the World Health Organization (WHO) Alma Atta Declaration of 1978 where the Primary Healthcare Approach prescribing the mobilization of untapped health human resources, in pursuing universal health coverage, was made [1]. In this endeavor, the Zimbabwean Ministry of Health (MoH) created the Health Services Board (HSB) in 2005, following the recommendation by a Presidential Review Commission of the Health Sector which had reported health-care worker challenges. Following this creation, however, health personnel problems worsened in 2007–08 as a result of the prevailing economic downturn. The Global Political Agreement that followed provided an avenue through which the pursuit of health worker reform was revived thereby resulting in the 2009 HRH Policy in Zimbabwe [1,6,7]. In the context of this, HRH in Zimbabwe is governed at two main levels, namely, the principal and agent levels as outlined in Figure 1. The principal level is the center and consists of Ministry of Health, and Health Services Board, and its deconcentrated field offices at provincial and district levels. In this, National HRH Taskforce with structures at national, provincial and district levels, consisting of the HSB, Provincial and District Medical Officers was set up in 2009. This Taskforce also consists of representatives from support Ministries (such as the Ministries of Finance, Local Government, Higher and Tertiary Education, and Foreign Affairs), Zimbabwe Association of Church Hospitals (ZACH) and program-specific donor organizations from the Zimbabwe United Nations Development Assistance Framework (ZUNDAF). The decision role of actors at this level is to formulate, support, regulate, supervise, monitor and evaluate the implementation of HRH reform policy [1,4].

The agent level is made up of a hierarchy of local governments consisting of city councils, municipal councils, town councils, and local boards. Local boards are the lowest in the hierarchy of urban councils. There are four such local boards established at Hwange, Ruwa, Epworth, and Chirundu. In this context, this study sought to use the Decision Space Approach to determine how the national human resources for health policy interventions of 2009 impacted the local health-care worker system of Epworth, a low-income peri-urban community in south-east Harare, Zimbabwe, and implications that this has towards the goal of the 2030 health-care worker reform agenda [1].

**Figure 2.**
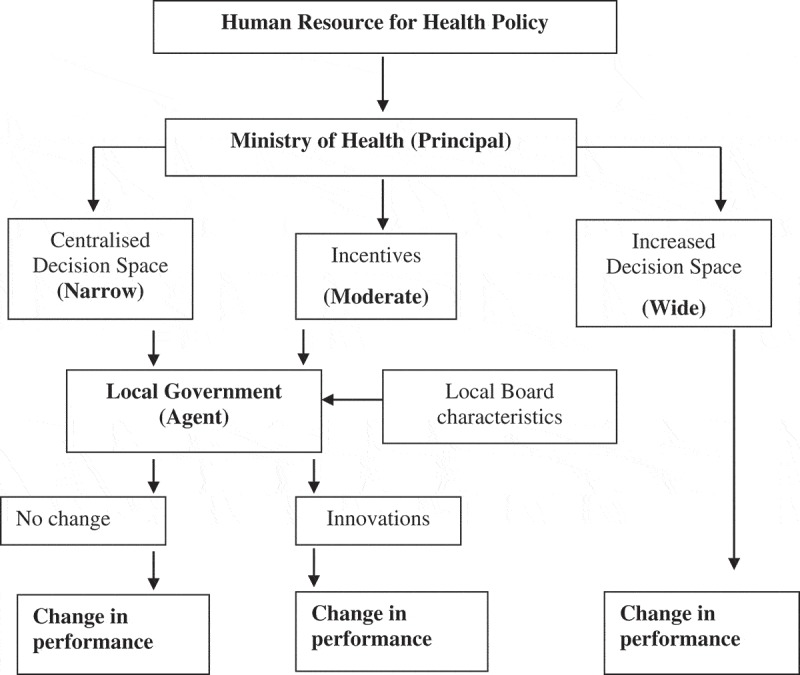
The decision space approach. Source: Bossert [9].

The Decision Space Approach shown in Figure 2 was developed by Dr. Thomas Bossert of the Harvard School of Public Health (HSPH) and used to facilitate analysis of the impact of decentralization of health systems with a focus on: (i) decision space between the principal and agent levels; (ii) innovation and (iii) performance [8,9]. Decision space is the range of effective choice (decision space/decision-making power or authority) allowed by the central authorities (the principal) to be utilized by local authorities (the agent) [1,4,8]. Decision space is characterized by the extent of decision-making which can either be wide (decentralized), moderate (shared) or narrow (centralized). Wide decision space is decentralized decision-making authority in which the local level is allowed total independence to innovate. Moderate decision space is conditioned decision-making authority in which the local level is influenced by incentives, defined rules and regulations, and parameters and sanctions. Actors may only also engage in functional innovation in which they make modifications and adjustments to suit local contexts. Narrow decision space is centralized decision space in which the principal (central government) retains all decision space over the local level. Decision space is determined by formal rules (laws and regulations, and national court decisions, administrative norms and standards, and the financial and technical capacity to enforce decisions), and informal rules (political rules of the game such as conformity to decision-making choices and interests of a certain authority). The actual (‘formal’ or ‘informal’) decision space may also be defined by lack of enforcement of these formal decisions [1,4].

The Decision Space Approach had been used in prior studies on health sector reform studies in Chile, Colombia, Bolivia, Zambia, Ghana, Uganda, Philippines and Fiji [8–10]. Whilst these studies used the Decision Space Approach to analyze health systems, the focus of salaries, contracts and civil service on health human resources provided a foundation upon which to take this forward towards the health worker reform agenda [4]. At the time that the Epworth study was carried out, the Pakistan study had been the only one to report the use of the Decision Space Approach to analyze the health worker function beyond salaries, contracts and civil service, by focusing on performance management, hiring, transfer, substitution, disciplinary actions, promotion, contracting and firing of health-care workers in decentralization-oriented reforms of 2001 [10]. Following completion of the Epworth study, two more similar studies have since been reported in Ghana and Uganda. The Ghana study analyzed decision space, innovation, and performance in recruitment, remuneration, and training and development [11]. The study in Uganda was closer to the Epworth study and focused on assessing decision space in six policy result areas, namely, policy, planning, remuneration and incentives, performance management, education and information [12]. These studies have helped broaden discussion in the endeavor to understand Decision Space Approach and what it can contribute towards understanding decision space, innovation and performance by principal and agent level actors in pursuing the health-care worker reform policy goal of universal health coverage in low- and middle-income communities [3–5].

## Methods

### Study location and context

The study was carried out in Epworth, a low-income peri-urban area on the south-east edge of Harare, Zimbabwe. Epworth is governed by a Local Board consisting of members elected by the community, who are responsible for managing the area including the collection of municipal rates and other levies. This peri-urban area is characterized by immigration, impoverishment and unplanned settlement on land without any water supply, sewer and sanitation systems in almost all parts of the community. The local management of human resources for health system falls under the Epworth Local Board, through a local governance process of devolution, and the Seke Medical District and the Mashonaland East Provincial Medical Directorate, deconcentrated offices of the Ministry of Health and Child Care [1]. This study site was also selected because of the limited research attention in the discussion on how decentralization of national health worker policy interventions impacts low-income peri-urban areas using the Decision Space Approach but yet these areas are the most affected by the global health workforce crisis as reported by the 2006 World Health Report [1–5]. Epworth is governed by a local board formed in 1986 in the context of the Urban Councils Act. Epworth consists of seven wards in which there are seven private clinics, two municipal clinics, and one mission clinic. Staff registers at each clinic revealed a total of 101 health workers of all cadres, excluding Medical Doctors and Sisters in Charge at health facilities across Epworth. A summative overview of the staff establishment is outlined in Table 1.

**Table 1.** Health worker numbers at clinics in Epworth.

Facility Type	Human resource for health managers	Nursing staff	Other cadres	Total for all cadres (excluding medical doctors and sisters in charge)
Mission clinic	**1** Sister in Charge	**2** Primary Counsellors; **6** Registered General Nurses; and **2** Primary Care Nurses.	**1** Environmental Health Officer/Technician; and **4** Nurse Aides.	**15**
Municipal ‘Polyclinic’ clinic	**1** Sister in Charge	**11** Registered General Nurses; **6** Midwives; **1** State Certified Nurse; **3** Primary Care Nurses; **2** Primary Counsellors.	**1** Pharmacy Technician; **3** Laboratory Scientists; **3** Ambulance Drivers; and **1** Environmental Health Officer; **11** Nurse Aides.	**42**
Municipal ‘OI’ clinic	**1** Sister in Charge	**13** Registered General Nurses.	**1** Dispensary Assistant; **1** Environmental Technician; **5** Nurse Aides; and **1** Pharmacy Technician.	**21**
Private clinic	**1** General Practitioner.	**1** Registered General Nurse; **1** Primary Care Nurse.	**2** Nurse Aides.	**4**
Private clinic	**1** General Practitioner.	**1** Registered General Nurse.	**3** Nurse Aides; **1** Lab Pathologist; **1** Radiologist; **1** Dental Surgeon.	**7**
Private clinic	**1** General Practitioner.	**1** Registered General Nurse; **1** Midwife.	**2** Nurse Aides.	**4**
Private clinic	**1** General Practitioner.	**2** Registered General Nurses.	**1** Nurse Aide.	**3**
Private clinic	**1** General Medical Practitioner	**1** Primary Care Nurse.	**0**	**1**
Private clinic	**1** General Practitioner.	**2** Registered General Nurses.	**0**	**2**
Private clinic	**1** General Practitioner		**2** Nurse Aides	**2**
**Total**		**56**	**45**	**101**

Reproduced with permission from BH Taderera [1,13].

In this table, it is shown that the Mission clinic had 15 health cadres, but there were no Medical Doctors at this facility. Two Municipal clinics in this community had a combined number of 63 health-care workers, 42 of whom were at one of them, and there were no Medical Doctors at these facilities. Private clinics in this area had a combined total of 38 health-care workers and seven general medical practitioners (medical doctors) in total [1,13].

### Research design

The research design was a case study in which cross-sectional studies were carried out at the principal and agent levels [14,15]. The Decision Space Approach was used to guide data collection and analysis [9]. Qualitative and quantitative data were collected from different sources that include policy documents, key informants, focus group discussion participants, and non-participant observation. The rationale behind this was to generate a comprehensive dataset for validity and reliability. Qualitative and quantitative datasets were integrated to enable cross-checking/comparison to identify potential data anomalies [15]. Data were collected during fieldwork between July and December 2015 [1].

### Qualitative study

Research started with a cross-sectional study at the principal level where a documentary search of the 2009 health human resources policy documents and strategic plans was carried out to explore policy goals, instruments and result areas. The documentary search generated an interview guide that was used in seven key informant interviews were carried out with seven purposively selected policymakers drawn from Ministry of Health (MoH), Zimbabwe Association of Churches (ZACH), Health Services Board (HSB), the Provincial Medical Directorate of Mashonaland East (PMDME), and the Seke District Medical Office (SDMO) to explore human resource reform policy context, policy result areas, decision space, innovation and performance. This interview guide was piloted in the first two of these seven key informant interviews. The interviews were face to face within which interview guides, notebooks, and audio digital recorders were used. The duration of these interviews was between 30 and 45 min, and data were collected until saturation was reached. Data collected at the principal level generated a Human Resources for Health Decision Space Mapping Analysis Conceptual Tool made up of six main policy result areas identified and shown in Table 2 [1,4].

**Table 2.** HRH decision space mapping analysis conceptual tool.

	Level of Decision Space		
Result Areas	Narrow	Moderate	Wide
**1. Human Resource Planning and Financing**			
● Demand and Supply Forecasting		MHCC;^a^ELB;^b^ Mission^c^	Private clinics
● HRH Financial Budgeting		MHCC; ELB; Mission	Private clinics
● HRH Strategic Partnerships	MHCC		
**2. Production, Training, and Development**			
● Capacity building for training critical HRH	MHCC; MHTE^d^		
● Support for further training	MHCC		Private clinics
● Centers of specialization	MHCC		
● Induction and exchange programs	MHCC		Private clinics
**3. Deployment, Retention, Utilization and Management**			
● Deployment		MHCC; ELB; Mission	Private clinics
● Retention and motivation		MHCC; ELB; Mission	Private clinics
● Performance management and utilization	MHCC		Private clinics
**4. Health Labour Relations**			
● Rights framework	MHCC		
**5. Health and Safety**			
● Health welfare			MHCC; ELB; Mission; Private Clinics.
● Safety and protection	MHCC		
**6. Human Resource Information and Research**			
● HRH Information System	MHCC		Private clinics
● HRH Research	MHCC		Private clinics

^a^MHCC is the Ministry of Health and Child Care through the Health Services Board.

^b^ELB refers to the Epworth Local Board, which is the local municipal (local government) authority in Epworth.

^c^Mission is a church-owned but government-run clinic. In this context, the mission was the Methodist Church Mission.

^d^MHTE refers to the Ministry of Higher and Tertiary Education.

The study then proceeded to the agent level in Epworth where data were collected through a cross-sectional study from 10 purposively selected local key informants that included community policymakers and health personnel managers (sisters in charge). The aim was to determine local decision space, functional innovation, and performance (health-care worker outcomes). The interview guide used in these local key informant interviews was piloted in the first two of these ten face-to-face interviews. Qualitative data were extracted from 21 purposively selected semi-structured questionnaires from sample interviews carried out health-care workers (these included nurses, nurse aides, primary counselors, environmental health officers, pharmacy technicians, laboratory technicians, and ambulance drivers) at local clinics to determine the impact of the 2009 HRH reform policy on health workers. These 21 interviews selected included two from the pilot study. In addition, five focus group discussions were carried out with community members. Participants were purposively selected and grouped into two main categories, namely, community health volunteers (Village Health Workers/Community Health Workers and Peer Educators) who participated in two focus group discussions, and ordinary community members who took part in the other three. Each of the five focus groups consisted of 10 participants, and data were collected to explore their role in the health-care worker reform process, healthcare service delivery outcomes, and the community health situation. The interview guide used was piloted in the first focus group discussion. In addition to the interview guide, a digital audio recorder, notebooks, and pens were used to facilitate data collection [1,14]. Qualitative data were collected in each category until saturation was reached and each interview lasted between 30 and 45 min [14]. An additional two focus group discussions were carried out with community members to explore potential areas of follow-up postdoctoral studies in this peri-urban community [1].

### Quantitative study

Numerical data were collected through a documentary search of staff registers at the local clinics to determine numerical changes in the number of health workers at each clinic. The number of health personnel was an important performance measure in this study given its centrality in the global health workforce crisis towards which this research seeks to help mitigate [1,15].

### Data analysis

#### Qualitative analysis of primary data

Qualitative data came from primary sources and were first transcribed, coded and then grouped into themes based on findings in the six health-care worker reform policy result areas identified. The focus of analysis was on the level of decision space, innovation (policy creativity) and performance (policy performance in terms of outcomes) in each of the six main result areas of the HRH Decision Space Mapping Analysis Conceptual Tool [1,4,8].

#### Quantitative analysis of desk review data

Quantitative data were generated from secondary sources through a documentary search and were presented numerically and tabulated focusing on changes in staffing levels. Descriptive statistics were used to support analysis of quantitative data, whilst interpretive thematic analysis was used for qualitative results. Qualitative and quantitative data were integrated to enable cross-checking and comparison for normality [14,15].

## Results

### Human resources for health decision space, innovation, and performance

Key informants and a documentary search at the p level showed that the 2009 HRH Policy and HRH Strategic Plan of 2009–2014 guided the health workforce reform intervention in Epworth [6,7]. Findings revealed six main policy result areas that included: Human Resource Planning and Budgeting; Recruitment, Training and Development; Deployment, Retention and Performance Management; Labour Relations; Health and Safety; and Human Resource Information Systems and Research shown in Table 2 [1].

Depending on the policy result area, decision space between the principal and the agent in the Epworth study was determined by rules and regulations, administrative norms and standards, financial and technical capacity and political rules of the game. For example, decision space in policy result areas that include demand and supply forecasting, financial budgeting, deployment and retention, and HRH information was determined by administrative norms and standards.

#### Human resource planning and budgeting

This reform policy result area sought to attain at least a balance in demand and supply of health-care workers through demand and supply forecasting, financial budgeting, and strategic partnerships. Human resource planning and budgeting were undertaken through collaborative and complementary decision space between the Epworth Local Board, and the Ministry of Health towards projecting the demand and supply of health personnel for the two Municipal clinics and one Mission clinic. This resulted from intervention through providing health cadres by the health ministry, necessitated by failure by the Epworth Local Board due to technical and financial incapacity, semi-formal settlement, an ever-increasing impoverished population, overwhelming disease burden, and the prevailing socio-economic challenges that had worsened during the 2007–8 period thereby crippling the local health-care worker system. In this shared decision space, the Ministry of Health also provided budget support through deconcentration towards salary payment for all the health cadres provided, whilst the Epworth Local Board and Church Mission complemented this effort and paid salary top-up allowances [1,4,13]. However, whilst planning reduced the gap between the demand and supply of health-care workers at the two municipal clinics and private clinic between 2009 and 2014, supply still fell short of requirements [4,13]. Key informants and a documentary search revealed that this emanated from principal level budget constraints that appeared to result from a low proportion of expenditure towards health from the national budget, which averaged 9, 63% of the total between 2009 and 2014 as shown in Table 3 [1]. Budget constraints at the principal level undermine the effect that narrow decision space can have at the agent level.

**Table 3.** Proportion of expenditure from the share of the national budget.

	Proportion of expenditure from share of national budget					
Year	2009	2010	2011	2012	2013	2014
Proportion of expenditure	8, 56%	8, 58%	9, 33%	8, 64%	10%	12, 7%

Source: BH Taderera [1].

This was compounded by agent level budget constraints which resulted in the local board and mission not being able to pay salary top-up allowances to all health workers at the three local public clinics [1, 16]. The local private sector enjoyed wide decision space in HRH planning, which was also limited by budget constraints as a result of a narrow revenue base that appeared to emanate from fewer clients in an impoverished community whose majority could not afford private medical care [4,13].

##### Strategic donor partnerships

The reform policy result area of strategic donor partnerships was meant to mitigate these challenges, through program-specific interventions by international health partners from the Zimbabwe United Nations Development Assistance Framework (ZUNDAF) [1]. Decision space was centralized because of political rules of the game within which consultation between the health ministry, Ministry of Foreign Affairs and international health donor partners at the principal level were informed by international health relations. Principal level key informants revealed that in these consultations, international health partners only came in to complement government effort in areas where there was technical and financial incapacity. In this context, a program-specific donor intervention on TB and HIV/AIDS that provided technical and financial support was implemented and contributed towards revival of the health human resource system as explained by one principal level key informant as follows:

*“The Ministry of Health through the Health Services Board played the role of identifying and establishing strategic partnerships with national, regional, continental and groupings on health personnel. This was done through the Ministry of Foreign Affairs. Where need be, and in the context of the Zimbabwe United Nations Development Assistance Framework, we engaged international health partners. However, you find that donors only come in as partners to complement government effort through relief aid or programme specific interventions in identified areas where technical and financial help is required (Key informant: 1)* [1].*”*

#### Training and development

Findings showed that this result area sought to strengthen capacity for training health workers, provide support for post-basic training, establish centers of specialization, and induction and exchange programs [1,4,16]. Decision space on capacity strengthening for training was determined by administrative norms and standards enshrined at the principal level, in the Ministry of Health training centers such as Parirenyatwa School of Nursing and Marondera Provincial Hospital, and the Ministry of Higher and Tertiary Education learning institutions such as Harare Polytechnic College and the College of Health Sciences, University of Zimbabwe. For Epworth, this resulted in a narrow decision space [1,16]. In this context, decision space towards the provision of support for post-basic training of health-care workers in Epworth was centralized. This level of decision space was determined by administrative norms and standards of the Ministry of Health which provided guidance on priority areas of training towards which support was provided and determined the number of health cadres from Epworth who would receive such support through its deconcentrated offices at provincial and district levels. Local key informants revealed that support for post-basic training included facilitated enrolment, paid study leave, and tuition fee waivers [16]. However, whilst some health-care workers appreciated this, others complained of limited post-basic training opportunities, and unavailability of funding options to enable pursuit of training in specialty areas of one’s choice. The Ministry of Health attempted to mitigate these challenges through the provision of regular in-house training workshops on various aspects of healthcare which somewhat helped reduce the knowledge gap. In addition, provincial-level participants revealed that there was centralized decision-making in the establishment of provincial-level centers of specialization, meant to be avenues through which specialist training would be provided, and induction and exchange programs for the sharing of competencies and beneficial work experience. However, whilst the technical and financial requirements resulted from centralized decisions, implementation was undermined by financial constraints [1,13,16].

#### Deployment, retention and performance management

Key informants revealed that the reform policy goals in this decision area were to deploy an adequate number of health cadres, retain them, and manage their performance. There was shared decision-making between the Epworth Local Board, mission church and the health ministry on deployment and retention of health workers, determined by financial and technical capacity. Incapacity by the agent resulted in intervention by the principal, thereby resulting in the increase in the total number of nursing staff from 17 in 2007 to 56 in 2014, 45 of whom were on the civil service payroll whilst the rest were employed by the local private sector. In addition, the number of other cadres (Nurse Aides, Environmental Health Officers, Primary Counsellors, Pharmacy Technicians, Dispensary Assistants, Pharmacy Technicians, Ambulance Drivers, Laboratory Scientists) increased from 7 in 2007 to 45 in 2014, 32 of whom were on the government payroll. In addition, about 30 Community Health Volunteers (Village Health Worker/Community Health Volunteer, and Peer Educators) were also recruited and deployed through complementary effort between the health ministry, local board and donor partner. The local board also enabled local private health sector participation thereby resulting in 11 out of the 56 nurses, and 6 out of the 27 nurse aides, and 7 general medical practitioners being contributed. Despite improved deployment, local health workers and community volunteers revealed that the number still fell short of requirements [1,13,16]. These shortages were also reported by community members who complained of long queues, congestion and the long time it took for them to be attended to at the clinic even though the situation had become better than before [1].

In order to retain the health cadres deployed, the Ministry of Health intervened to complement the local board and church mission through the timely payment of salaries denominated in USA Dollars. However, health workers complained that the salaries were inadequate for basic monthly needs. To mitigate this challenge, the local board and mission used their decision space to complement ministry effort through the payment of salary top-up allowances. However, health workers revealed that these salary top-up allowances were not paid to all health-care personnel which created a sense of division and exclusion that had a demoralizing effect on health-care cadres [1,16]. In order to manage the personnel performance in this reform context, the Ministry of Health adopted the Results-based Performance Management System as prescribed by the 2009 HRH reform policy. On the basis of administrative norms and standards, decision-making on this was centralized. However, implementation of this system was undermined by technical and financial incapacity at the principal level. Local key informants revealed that decision space on performance management was undermined by the local context characterized by staff shortages, and the visibly overwhelming workload in a high-pressure environment [1,13,16].

#### Legislation, health, and safety

In order to regulate the employment relationship, there were rules and regulations that determined the centralized nature of decision space in this result area. These included Section 29 of the Constitution of Zimbabwe, and Statutory Instrument 88B of 2005 from the Health Service Act (Chapter 15:16 No. 28/2004), Labour Act (Chapter 28:01), Public Health Act (Chapter 15:09), Medical Services Act (Chapter 15:13), and Health Professions Act (Chapter 27:19). However, legislation were irrelevant for management of health workers due to limitations and constraints of resources in the working environment [1].

Local public clinics enjoyed wide decision space regarding the health welfare of their health-care personnel within which they allowed their workers' liberty to seek medical aid cover (medical insurance) of their choice if need be. However, health workers revealed that they could not afford medical aid given their inadequate salary and the option to access free medical services in their workplace [1,4]. Health workforce safety and protection guidelines were however determined by the Ministry of Health, in conformity with World Health Organization requirements from which they were prescribed for implementation to local clinics in Epworth. The most common of these were the prophylaxis guidelines that helped protect health-care workers from accidental HIV/AIDS infection whilst performing their duties. However, whilst this provided a sense of protection amongst health-care workers, it appeared that the lack of knowledge and training amongst non-medical personnel, particularly Nurse Aides who worked in medical service areas undermined enforcement thereby resulting in accidental infection [1,16].

#### HRH information system and research

Principal level key informants and documentary search reported that the health ministry sought an HRH information system consisting of a computerized database, an observatory, with up to date information about the numbers, skills, caliber, and cadres of all health-care workers at each health facility in each district and province so as to help guide the HRH planning process. This was a new administrative norm and standard to be implemented at the Principal level by the health ministry. However, principal level key informants revealed that implementation was undermined by technical, financial and material incapacity thereby resulting in continued use of manual methods. Research was also aimed at providing data and information to support HRH reform policy decision-making. For Epworth, decision space was determined by financial and technical capacity of the district and provincial medical offices to support national-level HRH reform policy decisions [1,4].

## Discussion

The implications for decision space on innovation and performance of health-care worker reform policy in peri-urban areas are analyzed in four main categories (themes) that emerged from the study findings. The first theme is that moderate decision space enables the Principal (health ministry) to intervene in HRH Planning and Budgeting through a process of deconcentration to help mitigate local incapacities that undermine devolution and its contribution towards HRH reform policy in low-income peri-urban areas at the agent level. The Zimbabwe study shows that intervention by the principal resulted in a moderate limitation to decision space for the agent, but did enable mitigation of low incapacities. In this, findings show that moderate decision space allowed the agent to help mitigate financial and technical constraints of devolution in low-income peri-urban areas may help revive HRH planning and budgeting [1]. Even though desired outcomes may not be realized, this may help reduce the gap between demand and supply of health-care workers which, as reported in Epworth, has positive implications towards mitigating the global health workforce crisis in low-income peri-urban areas [2–4].

Narrow decision space provides an avenue through which to implement HRH reform policy interventions in peri-urban areas. This is the second theme from this study. First, the principal’s engagement of international health partners resulted in narrower decision space for the agent. This helped provide technical and financial support to help revive HRH reform policy intervention in low-income peri-urban areas [1–4]. On this, study findings show that the health ministry, in consultation with the Ministry of Foreign Affairs engaged international health partners from the Zimbabwe United Nations Development Assistance Framework (ZUNDAF) from which a program-specific donor intervention on TB and HIV/AIDS provided technical and financial support that also revived Epworth’s health human resource system [1]. Second, narrow decision space provides an avenue through which administrative norms and standards in strengthening capacity for training health workers may be upheld [1,4]. This is because low-income peri-urban areas do not have the capacity to strengthen the training of health-care workers. The Zimbabwe study revealed that such capacity is confined to higher learning institutions of the health ministry, and the Ministry of Higher and Tertiary Education such as the Parirenyatwa School of Nursing, Marondera Provincial Hospital, Harare Polytechnic College and the University of Zimbabwe. Thirdly, the principal (health ministry) also uses narrow decision space to uphold these administrative norms and standards through determining priority training areas towards which support is provided [1]. This helps improve the availability of competent health workers as prescribed by the Ouagadougou Declaration on Primary Healthcare and Health Systems in Africa, the 2018 Astana Declaration and the 2030 Global Workforce Strategy [3–5].

The third theme is that the principal may not always attain the desired performance outcomes by narrowing the decision space of the agent. On the one side, technical and financial constraints at the principal level undermine the implementation of innovative HRH reform policy decisions and the effect that they can have towards performance at the agent level. For example, the Uganda study reports that Human Resources Information Systems (HRIS) have emerged as an increasingly important result area facilitating the compilation and upkeep of up to date and comprehensive health workers’ personal files, including employment history, experience and skills acquired during training, information for analysis to guide health personnel reform policy decision-making at all levels [12]. However, lack of resources and technical capacity at the principal level may undermine the implementation of HRISR in low-income peri-urban areas as reported in Epworth [1]. There are also other varied reasons why narrow decision space may fail to achieve the desired impact. In performance management as an example, the Zimbabwe study reported the failure to fit the context as part of the reason behind the failure to adopt the newly prescribed results-based performance management framework [1,4]. Uganda and Ghana reported a similar finding in which it was revealed that performance management is undermined by lack of resources and the reluctance by frontline workers to be appraised as they do not see the benefits of it [12,16]. This has implications towards attainment of equity in distribution, availability, accessibility, competency, safety, motivation and information systems of health workers in low-income peri-urban areas [2,3,5]. On the other hand, the principal may lack the capacity to enforce implementation of choices in certain result areas at the agent level in low-income peri-urban areas. For example, the Epworth study suggests that health workforce safety and protection guidelines were difficult to enforce in high-pressure situations particularly amongst non-medical personnel such as Nurse Aides that provide a helping hand in those areas [1,16]. This undermines the goal of health-care worker safety, which in turn undermines motivation and retention of health personnel, and undermines healthcare delivery in these low-income areas [3,5].

The study in Zimbabwe shows that functional innovation to collaborate and complement each other in moderate decision space may not always result in desired performance levels in peri-urban areas. This is our fourth theme. For example, whilst health ministry intervention resulted in the deployment of more health-care personnel and payment of their salaries denominated in USA Dollars, the numbers still fell short of requirements in Epworth. More so, the salaries paid to health workers by the ministry were reported amongst health-care workers to be inadequate [1,4,16]. Further inquiry suggested budget constrains at the principal level due to a fall in the proportion of national budget allocated to health from share of national budget fell below the 15% minimum budgetary allocation requirement prescribed by the Abuja Declaration [1]. Budgetary constraints at the principal level may be compounded by financial incapacity at the agent level. For example, the Epworth Local Board and church mission failed to pay salary top-up allowances to all workers which created a sense of division and exclusion amongst health-care personnel which also has a demoralizing effect that undermines motivation and performance [1,16]. The strength of this study is that the discussion was based on primary and secondary data collected at both the principal and agent levels. However, there is room to explore how the level of decision space may change with time and implications that this has on HRH reform policy in low-income peri-urban areas.

## Conclusions

There is increasing importance in Decision Space Approach and what it can contribute towards understanding decision space, innovation and performance in the implementation of HRH reform policy interventions in resource-constrained peri-urban areas. In this endeavour, it is concluded that the Decision Space Approach can be used to analyze health worker reform policy interventions focusing on six main result areas that include: HRH Planning and Budgeting; Recruitment, Training and Development; Deployment, Retention and Performance Management; Labour Relations; Health and Safety and HRH Information Systems and Research. The Uganda study however also shows the importance of HRH Policy as a potential additional result area on its own. It is also concluded that the principal imposed moderate limitations on the decision space of the agent by intervening in HRH Planning and Budgeting through a process of shared decision-making to help mitigate local incapacities that undermine devolution and its contribution towards HRH reform policy in low-income peri-urban areas at the agent level. The Epworth study shows that even though desired performance levels may not be attained, this may help reduce the gap between demand and supply of health personnel which has positive implications towards mitigating the global health workforce crisis in low-income peri-urban areas. Narrow decision space may be used by the state may limit decision space further by applying political rules to engage strategic donor partners and to provide technical and financial support to help revive HRH reform policy intervention in low-income peri-urban areas, and provides an avenue through which administrative norms and standards in strengthening capacity for training health workers and determining priority training areas. However, it was also concluded that technical and financial constraints may undermine the narrow decision space which affects health information and research, and result in policy choices that do not apply to context particularly in performance management, and may be difficult to enforce in health-care worker safety. This affects the realization of HRH reform goals articulated by the 2018 Declaration of Astana, and the 2030 Global Workforce for Health Strategy in pursuit of universal health coverage in low-income peri-urban areas.

## Paper context

The Decision Space Approach presents an opportunity through which to address the global health workforce crisis in pursuing HRH goals stated by the Astana Declaration and the 2030 Global Health Workforce Strategy. To this end, this review synthesizes findings from the Epworth study and provides a new perspective by drawing from findings that have since emerged from similar studies in Ghana and Uganda to reinforce the pursuit of health-care worker reform.
